# Effects of Compressive Stockings and Standard Stockings in Skin Temperature and Pressure Pain Threshold in Runners with Functional Ankle Equinus Condition

**DOI:** 10.3390/jcm7110454

**Published:** 2018-11-21

**Authors:** David Rodríguez-Sanz, Ricardo Becerro-de-Bengoa-Vallejo, Marta Elena Losa-Iglesias, Eva María Martínez-Jiménez, Daniel Muñoz-García, Eduardo Pérez-Boal, César Calvo-Lobo, Daniel López-López

**Affiliations:** 1 Faculty of Sport Sciences, Universidad Europea de Madrid, 28670 Madrid, Spain; david.rodriguez2@universidadeuropea.es; 2 Facultad de Enfermería, Fisioterapia y Podología, Universidad Complutense de Madrid, 28670 Madrid, Spain; ribebeva@ucm.es (R.B.-d.-B.-V.); eva.hache2@hotmail.com (E.M.M.-J.); 3 Faculty of Health Sciences, Universidad Rey Juan Carlos, 28670 Madrid, Spain; marta.losa@urjc.es (M.E.L.-I.); perez.boal@gmail.com (E.P.-B.); 4 Departamento de Fisioterapia, Centro Superior de Estudios Universitarios La Salle, Universidad Autónoma de Madrid, 28023 Madrid, Spain; daniel.munoz@lasallecampus.es; 5 Motion in Brains Research Group, Instituto de Neurociencias y Ciencias del Movimiento, Centro Superior de Estudios Universitarios La Salle, Universidad Autónoma de Madrid, 28023 Madrid, Spain; 6 Nursing and Physical Therapy Department, Faculty of Health Sciences, Institute of Biomedicine (IBIOMED), Universidad de León, 24401 Ponferrada, Spain; 7 Research, Health and Podiatry Unit, Department of Health Sciences, Faculty of Nursing and Podiatry, Universidade da Coruña, 15403 Ferrol, Spain; daniel.lopez.lopez@udc.es

**Keywords:** ankle, foot, skin, thermography

## Abstract

Objective: To assess clinical differences in the Achilles tendons of runners with ankle equinus wearing either compressive or standard stockings. Design: Case–control study. Methods: In this study, we conducted clinical examinations of 98 sportsmen (runners) with equinus, before and after 30 min of running on a treadmill; 49 runners wore compressive stockings and 49 wore standard stockings. Clinical assessments of the runners’ Achilles tendons were based on the pressure pain threshold (PPT) and skin temperature analysis. Results: Achilles tendon evaluations identified significant differences in skin temperature modification and PPT between the compressive and standard stocking groups. Conclusions: Based on our findings, we propose that higher skin temperatures are associated with lower pressure pain thresholds in the Achilles tendons of runners with ankle equinus.

## 1. Introduction

Equinus is a clinical limitation of the range of dorsiflexion in the foot and ankle complex. Equinus can be defined as “dorsiflexion limitation of the ankle with the knee extended/flexed (excluding osseous restriction)” [1,2]. Although equinus is a non-symptomatic condition, it may promote clinical alterations in the Achilles tendon (AT) and triceps surae muscle. Equinus is significantly related to lower limb injuries (e.g., anterior cruciate ligament rupture), asymmetric loading patterns, and alterations in triceps surae contraction [3,4,5,6].

A lack of adequate ankle dorsiflexion can result in compensation within the gait cycle, such as an early heel lift and an increase in forefoot pressures [7], which causes pain in the forefoot [8].

The gastrocnemius muscle, in particular, is the predominant deforming force in people with structural breakdown or chronic pathological changes related to the foot and ankle. We suspect that contracture of this muscle is not only common but often partially responsible for many of the chronic forefoot and midfoot symptoms identified in non-neurologically-impaired patients [9].

Equinus can promote higher activation of the lower limb musculature. This clinical condition has been extensively studied using pressure platforms [10,11,12,13], and relationships between muscle contraction patterns in equinus have been investigated by electromyography [14,15]. Equinus is significantly associated with gait and posture [12,13], and with sports-related injuries. Activation of the skeletal musculature could modify the tendon’s mechanosensivity, and also promote heat transfer with a cutaneous temperature increase [16,17,18,19].

By using an infrared (IR) ThermaCam and an algometer, we aimed to check cutaneous temperature and pressure pain threshold (PPT) variations in runners with equinus condition, and also to examine the associations between the use of compressive stockings and standard stockings with skin temperature status and PPT values. The main purpose of this research was to assess thermal differences and PPT values in runners with equinus who were wearing either compressive or standard stockings before and after running.

## 2. Methods

This was an observational study. Ninety-eight healthy male subjects (runners) were recruited. A consecutive sampling method was chosen to select participants from an athletic running club. Forty-nine subjects wore compressive stockings, and 49 wore standard stockings. All subjects successfully completed this observational study. The exclusion criteria consisted of several parameters: (1) the presence of scoliosis, sprains, bone alterations, infections, and musculoskeletal and tendon injuries in the lower limbs; (2) low back or pelvic pain; and (3) use of drugs in the week prior to the assessment. 

The compressive stockings were made of 77% polyamide, 13% elastane, and 10% polyester, and covered the area from the foot to the inferior pole of the kneecap with graduated pressure; pressure was highest at the foot and the malleolus, and decreased proximally from 25 to 20 mmHg.

The variables studied included skin temperature and PPT. An IR ThermaCam (FLIR, Wilsonville, OR, USA) was used to register the temperature values of the Achilles tendon. PPT was measured with an algometer.

The sample size calculation was taken as the difference between two independent groups using the G*Power (Universität Düsseldorf, North Rhine-Westphalia, Germany) statistic tool, and was based on the right Achilles tendon PPT from a case–control pilot study with two groups (mean ± standard deviation (SD), *n* = 10 subjects with equinus condition (1.60 ± 0.57 kg/cm^2^) and *n* = 10 subjects without equinus condition (1.98 ± 0.57 kg/cm^2^)). Based on the one-tail hypothesis, an effect size of 0.67, α error of 0.05, and power of 0.95 were used for the sample size calculation. Therefore, a total sample size of 98 subjects, with 49 in each group, was obtained.

The Ethics Committee of the HULP (Madrid, Spain; record no. CE 2828A) approved the study. All subjects provided informed consent before the beginning of the study. The Declaration of Helsinki was respected. Strengthening the Reporting of Observational Studies in Epidemiology (STROBE) guidelines were applied.

Subjects were given standard protocol-related advice before attending the experimental assessment [16]. During the week prior to the trial, subjects were not allowed to use drugs. On the day of assessment, vasomotor substances (e.g., caffeine) and heavy meals were not allowed. An acclimatization period of 10 min in the examination room was completed for all the participants. Images were taken in the morning from 9:00 to 10:30 a.m.

First, participants lay in a supine position and the status of their equinus was assessed with the knee extended and flexed. Range of movement was assessed using a goniometer to check the angle between the plantar line of the foot and the tibia bone axis. PPT was checked using an algometer before and after 30 min of running. Equinus and PPT assessments were carried out by the same podiatrist in order to ensure the reliability of measurements. PPT was assessed with an analogue pressure-algometer (Mechanical algometer, FDK/FDN series Force-Dial, Wagner/Instruments, 1217-Greenwich, CT 06836; 0–10 kg/cm^2^). PPT was assessed on a scale ranging from 0 to 10 kg/cm^2^. Three measurements were repeated, with a 30- to 60-sec interval between measurements, and the mean of the three measurements was recorded. The algometer tip was situated over the Achilles tendon (6 cm up from the Achilles tendon insertion), and pressure was applied until the subject indicated that the sensation switched from pressure to pain. All PPT assessments were carried out after the thermal evaluation.

The first assessments for thermal image recordings and PPT measurements were carried out prior to participants putting their stockings on. Afterwards, participants put on their stockings (either compressive or standard) and began running. After completion of the activity, subjects removed their socks, and thermal images were recorded prior to PPT evaluation. The timeline for the subjects consisted of several steps: (1) entering the examination room; (2) undressing; (3) acclimatization; (4) thermal image recordings; (5) PPT assessments; (6) putting on stockings (compressive or standard); (7) treadmill running; (8) stocking removal; (9) thermal image recordings; and (10) PPT assessments.

An acclimatization period of 10 min in the examination room was completed by all of the participants. The infrared imaging examination (high-resolution thermogram analysis) started with the runner standing up in a relaxed position. The Achilles tendon region was measured five times with the FLIR ThermaCam. All measurements were acquired in a laboratory with a temperature of 24.1 ± 1 °C, humidity of 45% ± 10%, and no direct ventilation flow toward participants or raters. In order to improve the research quality, different paper signals were used to set the limits for the Achilles tendon with a rectangular shape (2 cm wide, 6 cm long). Subjects then ran for 30 min on a treadmill, at a speed of 10 km/h, and the IR imaging was repeated. 

Imaging was performed using an FLIR/SC3000/QWIP ThermaCam infrared thermal device with an 8–9 µm spectral range and a temperature sensitivity of 0.02 K (NETD at 30 °C). The 320 × 240 FPA device presents a 20° lens. Images were captured with a 1.1 mrad spatial resolution. IR image acquisition was carried out by the same clinician, using a tripod system, in order to ensure the reliability of measurements.

**Figure 1:**
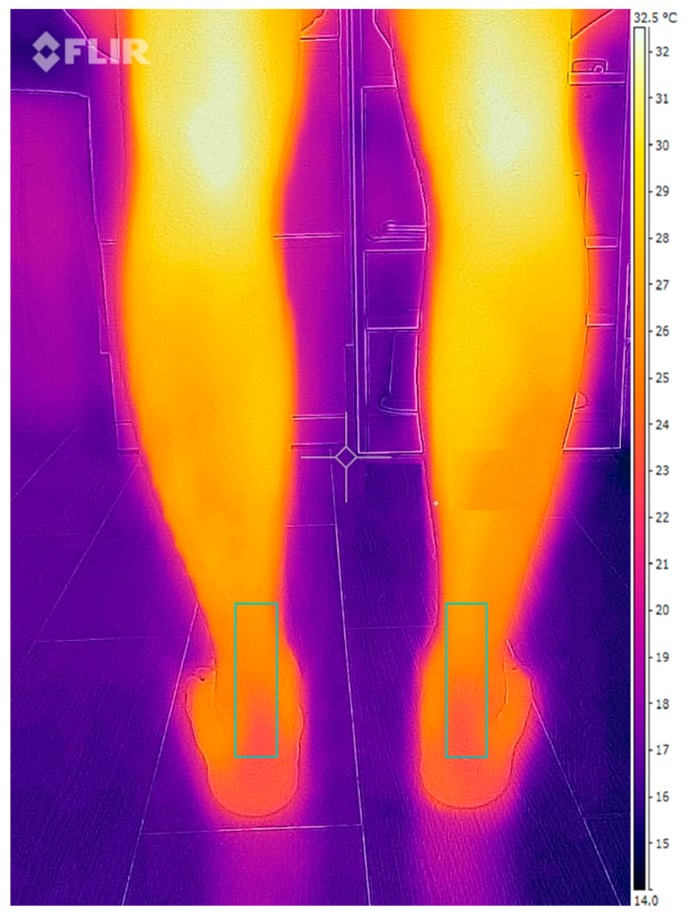
Achilles tendon infrared (IR) imaging.

IR images and data were evaluated with the FLIR^®^ software ThermaCam Researcher Professional V.2.9 (FLIR, Wilsonville, OR, USA). This software provides raters with different components of thermal values from the selected region. An IR imaging example is shown in Figure 1. The checklist for thermographic imaging in sports and exercise medicine (TISEM) was completed for reporting the conditions for thermal imaging [20].

Statistical analyses were supported by SPSS (version 22.0 for Windows, IBM SPSS Statistics for Windows, IBM Corp, Armonk, NY, USA) with an α error of 0.05 (95% confidence interval (CI)) with the desired power of 80% (β error of 0.2).

The Kolmogorov–Smirnov test was performed to check data normality. All data were normally distributed, and parametric statistical tests were selected. The mean and standard deviation of the temperature values and the PPT data were obtained for the Achilles tendon.

An unpaired Student’s t-test was performed to test for statistically significant differences in height, weight, body mass index, and age between the two groups. A paired Student’s t-test was performed to determine differences between the groups (compressive and standard stockings) and between imaging sessions (before and after running). 

## 3. Results

We found no statistically significant differences between the compressive and standard stocking groups with respect to participants’ heights, weights, ages, or body mass indices (Table 1).

We found no significant differences in Achilles tendon temperatures between the two groups before running (Table 2).

However, after 30 min of running, temperatures (maximum and mean values) were significantly higher in the standard stocking participants than in the compressive stocking participants (*p* < 0.05) (Table 3).

We did not find significant differences for the PPT measurements between the two groups before running. On the other hand, we found significant differences (*p* = 0.01) in the Achilles tendon PPT measurements between the group wearing compressive stockings (3.6 ± 0.31) and the group wearing standard stockings (2.9 ± 0.12) after 30 min of running.

## 4. Discussion

We identified an increase in maximum and mean Achilles tendon temperatures after exercise in subjects with standard stockings compared to those with compressive stockings. The minimum temperature values were also higher with standard versus compressive stockings, but these differences did not achieve statistical significance. The PPT values were lower with standard stockings than with compressive stockings.

The Achilles tendon undergoes greater contraction when running than when at rest, and therefore might be affected early by fatigue, thus explaining our observed increase in Achilles tendon temperatures in standard stocking subjects compared with compressive stocking subjects. When participants remained at rest [21,22], we did not find significant differences. Physical activity may serve as a stimulus for modifying temperatures in the lower limb muscles.

Compressive stockings may assist with a wider range of ankle movement during loading and running, and promote venous return. Biomechanically, the maximum ankle dorsiflexion during the stance phase of a normal gait occurs before heel lift, with the knee completely extended [1]. In the reviewed literature, static evaluation of ankle dorsiflexion range of movement shows that the minimum dorsiflexion movement for the ankle for normal gait is 10° of motion [3,21,22,23,24].

Additional studies will be needed to improve our knowledge of this muscle condition and to establish the clinical relevance of the association between temperature and PPT in non-equinus subjects. Based on our findings, we propose that compressive stockings could be recommended to runners with equinus.

## 5. Conclusions

Standard stocking participants had higher mean and maximum Achilles tendon temperature values after 30 min of running than compressive stocking participants. The standard stocking group also showed a lower PPT. Skin temperature measurements obtained by infrared thermography could serve as a complementary description of treadmill running for athletes with an equinus foot. Based on our findings, we propose that compressive stockings could be recommended for runners with gastrocnemius-soleus equinus condition.

### Practical Implications:

- Measurable skin temperature differences exist between sportsmen with equinus wearing compressive versus standard stockings after running. Further research is needed to understand the relationship between skin temperature at the Achilles tendon and the functionality of the triceps surae muscle in running athletes.
- After running, measurable PPT differences exist in the Achilles tendons of sportsmen with equinus wearing compressive versus standard stockings. Whether compressive stockings are potentially protective against Achilles tendon injuries warrants further research.
- Although compressive stockings may be advantageous in terms of modifying calf muscle function and providing Achilles tendon protection, there is insufficient evidence to favour either type of stocking for sport and recreational activities in runners with equinus.

## Figures and Tables

**Table 1:** Participant characteristics (*n* = 49 compressive stocking and 49 standard stocking participants).

	Compressive Stocking Group	Standard Stocking Group
Age (years) *	33.56 ± 3.61	35.2 ± 2.3
Height (cm) *	171.5 ± 5.1	169.1 ± 4.1
Weight (kg) *	71.2 ± 2.3	70.1 ± 2.1
Body mass index *	21.1 ± 1.3	20.8 ± 1.5

* No statistically significant difference between groups (*p* ≥ 0.05).

**Table 2:** Temperature values (degrees Celsius) for Achilles tendons for compressive stocking and standard stocking participants before exercise.

Variable	Mean	SD	*p*-Value
Achilles tendon (left) minimum temperatures before running			
Compressive stockings	26.52	±3.59	0.581 *
Standard stockings	27.22	±1.16	
Achilles tendon (left) maximum temperatures before running			
Compressive stockings	29.92	±1.58	0.19 *
Standard stockings	28.93	±1.75	
Achilles tendon (left) mean temperatures before running			
Compressive stockings	28.57	±1.92	0.393 *
Standard stockings	27.87	±1.47	
Achilles tendon (right) minimum temperatures before running			
Compressive stockings	26.79	±2.34	0.213 *
Standard stockings	26.85	±1.26	
Achilles tendon (right) maximum temperatures before running			
Compressive stockings	29.57	±1.88	0.324 *
Standard stockings	29.51	±1.13	
Achilles tendon (right) mean temperatures before running			
Compressive stockings	28.29	±1.68	0.48 *
Standard stockings	28.46	±1.93	

* No statistically significant difference between groups before running (*p* ≥ 0.05). *n* = 49 compressive stockings and *n* = 49 standard stockings; SD, standard deviation.

**Table 3:** Temperature values (degrees Celsius) for Achilles tendons in compressive stocking and standard stocking participants after exercise.

Variable	Mean	SD	*p*-Value
Achilles tendon (left) minimum temperatures after exercise			
Compressive stockings	31.04	±3.34	0.146 *
Standard stockings	30.75	±1.24	
Achilles tendon (left) maximum temperatures after exercise			
Compressive stockings	33.74	±2.02	0.019 ^†^
Standard stockings	31.1	±0.93	
Achilles tendon (left) mean temperatures after exercise			
Compressive stockings	29.3	±2.13	0.02 ^†^
Standard stockings	31.67	±1.3	
Achilles tendon (right) minimum temperatures after exercise			
Compressive stockings	31.41	±3.15	0.216 *
Standard stockings	30.85	±1.65	
Achilles tendon (right) maximum temperatures after exercise			
Compressive stockings	31.6	±1.79	0.04 ^†^
Standard stockings	33.9	±1.02	
Achilles tendon (right) mean temperatures after exercise			
Compressive stockings	29.1	±1.67	0.001 ^†^
Standard stockings	31.85	±1.2	

* No statistically significant difference between groups before running (*p* ≥ 0.05). ^†^ Statistically significant difference between groups (*p* < 0.05). *n* = 49 compressive stockings and *n* = 49 standard stockings; SD, standard deviation.
